# Path2Space for the AI era: enabling large-scale spatial cancer biomarker discovery from routine H&E images

**DOI:** 10.1186/s43556-026-00624-z

**Published:** 2026-11-11

**Authors:** Yihao Zhu, Shuangzhou Peng, Jin Ding

**Affiliations:** Clinical Cancer Institute, Center for Translational Medicine, Naval Medical University, 800 Xiangyin Road, Shanghai, 200433 China

In a recent paper published in *Cell*, Shulman and colleagues present Path2Space [1], a deep-learning model that predicts the spatial expression of 14,068 genes directly from routine hematoxylin-and-eosin (H&E) whole-slide images of breast cancer. Path2Space provides a cost-effective and scalable computational framework that supports cohort-level dissection of the tumor microenvironment (TME) and prioritization of spatial biomarkers.

Spatial transcriptomics (ST) preserves the positional information of gene expression within tissue architecture and links TME structure to tumor progression and therapeutic response [2]. However, its cost and limited throughput restrict it to small cohorts. By contrast, routine H&E images are abundantly available in clinical cohorts, raising the possibility of inferring spatial expression from them.

Prior methods have enabled H&E-to-spatial-expression interference at the tissue or spot level, but the vast majority covered only a small panel of genes, with limited clinical translational value. Path2Space overcomes this by predicting the whole-genome spot-level expression and applying it to cohort-level analyses. These inferred features were further shown to predict patient survival and treatment response more accurately than conventional bulk-sequencing biomarkers, at a cohort scale that individual ST studies cannot reach. This capacity unlocks large retrospective archives for spatial biomarker studies, an opportunity that prior work has left largely unexplored.

Foundational models, pretrained self-supervised on millions of histopathology tiles across diverse tissues, have provided well-established and generalizable solutions for analyzing histopathological images. Path2Space is not itself a foundation model, but a task-specific deep learning model that uses a one as a feature extractor. It predicts the spatial expression of 14,068 genes from H&E by encoding each image tile into 768-dimensional feature vector using the CTransPath foundation model and mapping these to expression values with a multi-layer perceptron (Fig. 1a). The model was trained on 56,567 matched image-expression spot pairs generated with the 10 × Visium breast cancer cohort of Bassiouni et al. (*n* = 14) under leave-one-patient-out cross-validation, with log-transformed counts as the prediction target and neighbor averaging for spatial smoothing. Across three independent ST cohorts including HEST (*n* = 4), Martinez et al. (*n* = 4), and HTAN (*n* = 7), 4,807 genes achieved a Pearson correlation above 0.4 in both cross-validation and external validation. The model also generalized from fresh-frozen to formalin-fixed paraffin-embedded slides and compared favorably with 16 other state-of-the-art predictors. Its inferred expression yielded cancer, lymphocyte, and stromal cell fractions from PanopTILs annotations that approached the accuracy of deconvolution from measured ST on external slides.

**Fig. 1:**
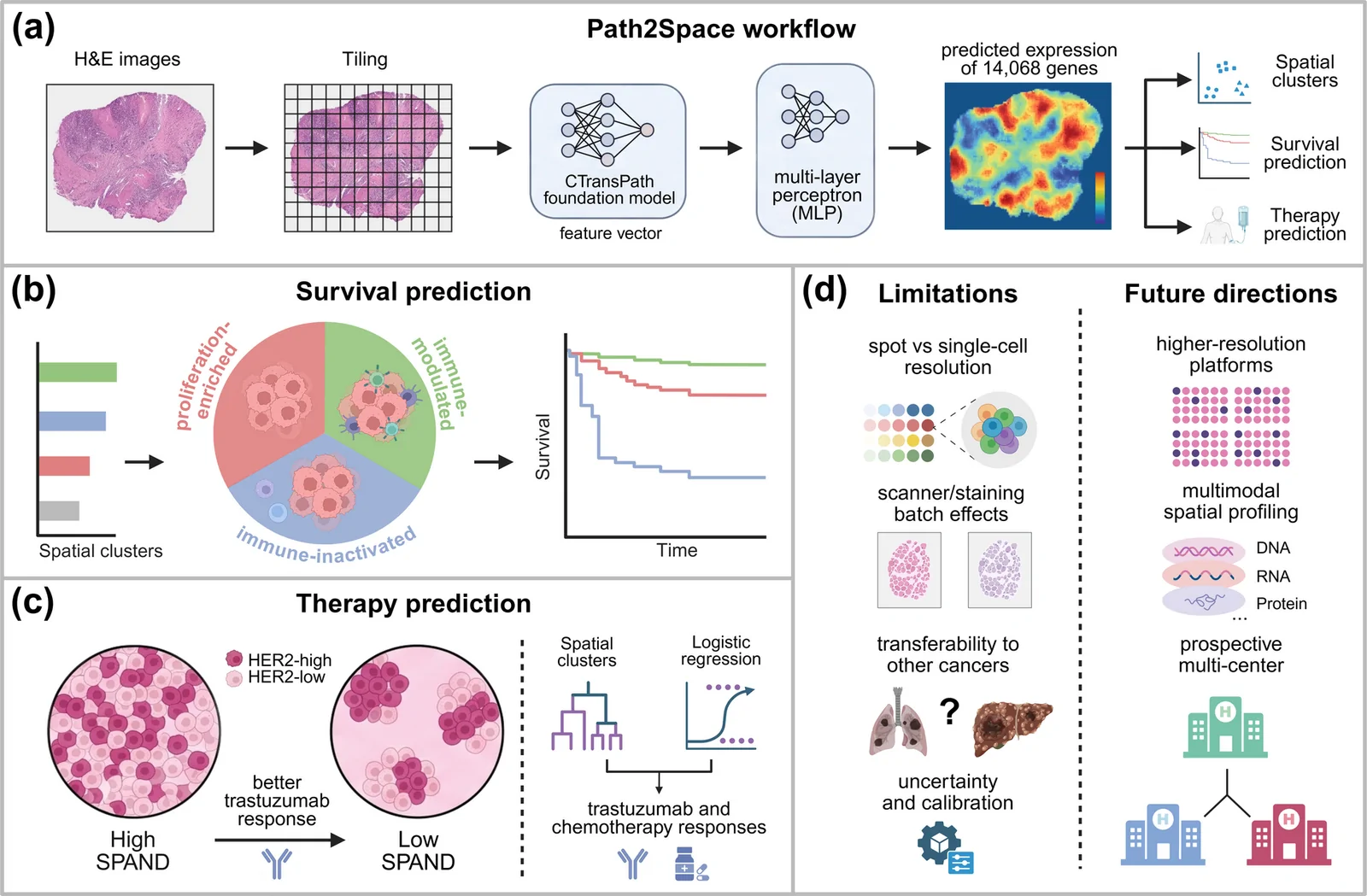
Path2Space enables large-scale spatial cancer biomarker discovery from routine H&E images. **a** Path2Space workflow; **b** Path2Space-inferred SpatioTypes predict TME composition and survival outcomes; **c** Path2Space-inferred SPAND metric and spatial clusters predict chemotherapy and trastuzumab response; **d** Limitations and future directions

Path2Space was then applied to discover spatial biomarker of survival and therapy response in larger cohorts (Figs. 1b-c). By analyzing 1,096 H&E slides from 976 TCGA patients (853 with survival data), Path2Space stratified ST clusters into three spatial subtypes (named SpatioTypes), including proliferation-enriched, immune-modulated, and immune-inactive subtypes. The immune-inactive subtype (n = 218) showed the poorest disease-free survival after adjustment for age and stage (HR = 2.04), validated in the METABRIC cohort (*n* = 141).

For HER2+ breast cancer, spatial neighborhood diverseness (SPAND) quantified the spatial heterogeneity of inferred HER2 expression within 100 × 100 µm neighborhoods, and tumors in which HER2-high and HER2-low regions intermix responded better to trastuzumab across four treated cohorts (TransNEO, PBCP, IMPRESS, and Cedars-Sinai). Where matched Fluorescence in situ hybridization (FISH) was available, HER2 SPAND was comparable to HER2/CEP17 FISH overall and more accurate in high-amplification tumors. SPAND is computed from inferred rather than measured HER2 expression, these findings are therefore limited to retrospective HER2+ cohorts and research use, and they do not equate the Path2Space-inferred output with FISH, immunohistochemistry, or measured ST data. Logistic regression models built from ST clusters also showed excellent cross-validation and external-validation performance for both chemotherapy and trastuzumab responses, outperforming published predictors.

Path2Space assumes that transcriptional states are sufficiently encoded in histomorphology and that this mapping transfers across settings. Therefore, it may fail for genes whose spatial expressions do not strongly correlate with tissue morphology or is not captured spatial transcriptomics. Transfer is also unlikely to hold uniformly across cancer types, clinical centers, scanners, staining protocols, and Visium resolution (Fig. 1d). Path2Space was trained on public breast cancer Visium data, its performance in real-world cohorts and in other cancer types requires rigorous multi-center validation. In routine practice, H&E images vary across scanners, staining protocols, and tissue fixation [3], and such domain shift may degrade predictions under specific scanning conditions. Patient-level cross-validation reduces these, but does not eliminate the risk that shared preparation pipelines or overlapping patients introduce center, specimen-processing, or data-leakage confounding that overstates generalization. Domain adaptation techniques are needed before clinical deployment. Path2Space additionally inherits the resolution of its training Visium platform, in which each spot corresponds to a 55-µm-diameter area that contains multiple cells, so outputs are spot-level rather than single-cell predictions and cannot resolve rare cell types. This is an inherent property of the training data rather than the predictive architecture, and higher-resolution ST platforms such as Stereo-seq could alleviate it [4].

Three further limitations warrant emphasis. First, predicted expression is evaluated against measured ST datasets, which themselves carries technical noise from variable capture efficiency and dropout, so the ground truth is imperfect. Second, spatial smoothing can blur boundaries between adjacent tissue compartments. Third, the model outputs estimate without calibrated uncertainty, limiting confidence in individual predictions.

Given these limitations, virtual predictions from Path2Space should not be regarded as substitutes for measured ST data. We believe the greatest near-term value of Path2Space lies in research applications, where virtual spatial transcriptomics can be layered onto existing H&E archives for hypothesis generation and spatial biomarker prioritization. Compared with multimodal foundation models, Path2Space’s advantage is practical and scalable implementation. Because it performs no cross-modal pretraining and learns its image-to-transcriptome mapping by supervised regression on paired data, it remains modular scalability and computationally light. It can be migrated to a new cohort or a new cancer type at lower cost, which makes it easy to deploy in research applications. The trade-off is that it does not inherit the cross-tissue and zero-shot transfer abilities that those multimodal models acquire through pretraining, so each new setting still requires paired training data.

Large-scale TME atlases are measured, cell-type-resolved reference maps of the same molecular modality that Path2Space predicts, and they serve as its ground truth. Spatial-proteomic imaging differs from Path2Space by modality rather than by panel size. These platforms resolve tens to more than 100 proteins at subcellular resolution, but they require dedicated sections and instruments, and their proteome coverage remains incomplete. Path2Space-predicted expression can therefore augment measured spatial information without additional experimental burden. Since co-profiling spatial transcriptome and proteome in the same tissue section remains technically demanding and costly [5], this may offer a practical route toward multimodal spatial profiling in digital pathology workflows. Nevertheless, clinical deployment requires prospective multi-center validation and rigorous comparison with well-established assays.

Overall, Path2Space provides a scalable and low-cost strategy for predicting ST from routine H&E images. We anticipate that Path2Space can be adapted to additional cancer types and subject to prospective multi-center validation, translated toward clinical applications that guide more personalized diagnostic and treatment decisions.

## Data Availability

Not applicable.
